# Independent Associations Between Arginine-Related Metabolites and Disease Activity in Pediatric Asthma: A Multivariable Biomarker Study

**DOI:** 10.3390/biom16050631

**Published:** 2026-04-24

**Authors:** Emine Aylin Şenol, Öner Özdemir, Aysel Özpınar, Muhittin A. Serdar

**Affiliations:** 1 Department of Biochemistry and Molecular Biology, Graduate School of Health Sciences, Acibadem Mehmet Ali Aydinlar University, 34752 Istanbul, Türkiye or aylin.yilmaz@cbu.edu.tr (E.A.Ş.); aysel.ozpinar@acibadem.edu.tr (A.Ö.); 2 Department of Pediatric Neurology, School of Medicine, Manisa Celal Bayar University, 45140 Manisa, Türkiye; 3 Department of Pediatric Allergy and Immunology, School of Medicine, Sakarya University, 54187 Sakarya, Türkiye; oner.ozdemir.md@gmail.com; 4 Department of Medical Biochemistry, School of Medicine, Acibadem Mehmet Ali Aydinlar University, 34752 Istanbul, Türkiye

**Keywords:** asymmetric dimethylarginine, symmetric dimethylarginine, asthma, exacerbation, children

## Abstract

Asthma is a heterogeneous inflammatory airway disease with variable clinical phenotypes. Dysregulation of the arginine–nitric oxide (NO) pathway contributes to airway hyperresponsiveness and endothelial dysfunction, but its role across stages of pediatric asthma remains unclear. In order to replicate real-world clinical heterogeneity, this investigation assessed serum levels of L-arginine, symmetric dimethylarginine (SDMA), asymmetric dimethylarginine (ADMA), L-citrulline, and the SDMA/ADMA ratio in children with managed asthma, asthma exacerbation, upper respiratory tract infections (URTIs), pneumonia, COVID-19, and healthy controls. Discovering stage-specific arginine pathway changes and evaluating their ability to distinguish asthma and asthma exacerbations from other clinically similar respiratory disorders was the primary aim of our research study. Receiver operating characteristic (ROC) analysis demonstrated that the SDMA/ADMA ratio achieved the strongest individual discriminative performance for distinguishing asthma exacerbation from controlled asthma (AUC: 0.917), while the combined multimarker model incorporating all four metabolites yielded an AUC of 0.983 with a sensitivity of 87.5% and specificity of 95.5%. These results indicate that arginine pathway metabolites, especially the SDMA/ADMA ratio, may merit additional research as possible markers of acute disease activity in asthma, even though they are exploratory and need external confirmation.

## 1. Introduction

Asthma is a heterogeneous disease rather than a single clinical entity, distinguished by episodic airway obstructions, hyperresponsiveness, and a variety of phenotypic characteristics [1]. Disease manifestations may be triggered by exercise, exposure to allergens or irritants, environmental changes, and viral respiratory tract infections. In a subset of patients with asthma, worsening lung function has been associated with increased arginase activity, which reduces the bioavailability of arginine, the primary substrate for nitric oxide synthase (NOS) isoforms. Consequently, diminished arginine availability impairs NO-dependent signaling, reduces S-nitrosothiol formation, and promotes bronchoconstriction.

Nitric oxide (NO) is a free radical that plays a significant role in regulating airway tone, maintaining the balance between arterial and venous circulation, and modulating immune system function [2]. The NOS enzyme family produces NO by converting the terminal guanidino group of L-arginine to NO [3]. ADMA, an endogenous arginine analogue, functions as a competitive inhibitor of NOS and inhibits cellular L-arginine uptake and NO synthesis [4,5]. Increasing clinical and experimental evidence shows that elevated ADMA levels are associated with oxidative stress, inflammation, endothelial dysfunction, atherosclerosis, apoptosis, autophagy, and immune system dysfunction, highlighting the role of ADMA as a significant risk indicator [6,7,8].

**Figure 1:**
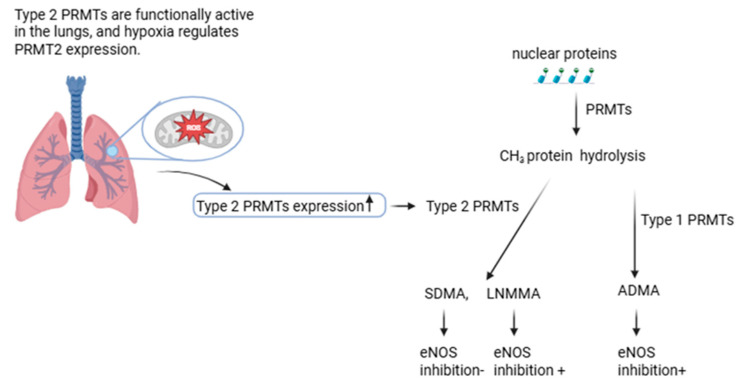
The regulatory effects of hypoxia on arginine residues through PRMTs [10,14]. ADMA: asymmetric dimethylarginine, LNMMA: NG-monomethyl L-arginine, PRMTs: protein arginine methyltransferases, SDMA: symmetric dimethylarginine. Figure was created by the Authors.

ADMA is generated through post-translational methylation of arginine residues by type 1 protein arginine methyltransferases (PRMTs), whereas type 2 PRMTs produce SDMA, and its monomethylation results in the formation of LNMMA [9]. While SDMA does not directly inhibit NOS, both ADMA and LNMMA contribute to endothelial dysfunction via competitive inhibition of NOS [5,10,11,12]. Notably, type 2 PRMTs are functionally active in the lungs, and they are found in the presence of hypoxia commonly encountered during acute asthma exacerbations, and they have been shown to regulate expression of PRMT2 and concentrations of pulmonary ADMA (Figure 1) [10]. In pediatric asthma, characterized by chronic airway inflammation, oxidative stress, and intermittent hypoxia, dysregulation of ADMA metabolism may therefore play a critical role in impaired bioavailability of NO, airway hyperresponsiveness, and disease severity [10,13,14].

Unlike traditional case–control study designs, this study was designed to replicate real-world clinical settings by including both different stages of asthma and other respiratory disorders with overlapping symptoms. Its primary goal was to evaluate L-arginine, SDMA, ADMA, and L-citrulline levels and the SDMA/ADMA ratio between children with controlled asthma and those with asthma exacerbation(s) to identify stage-specific metabolic alterations. Additionally, by incorporating children with upper respiratory tract infection (URTI), pneumonia, and COVID-19, as well as healthy controls, the study aimed to assess whether these changes are asthma-specific or components of a broader inflammatory response. It also aimed to evaluate the potential of these biomarkers to distinguish asthma and asthma exacerbations from other clinically similar respiratory conditions.

## 2. Materials and Methods

### 2.1. Study Participants

This study was designed as a comparative, cross-sectional analysis. The primary comparison was conducted between children with controlled asthma (n = 22) and those with asthma exacerbation(s) (n = 24) to evaluate changes in arginine-related metabolites across different clinical stages of the same disease. In addition, three disease control groups, URTI (n = 38), pneumonia (n = 15), and COVID-19 (n = 36), as well as healthy controls (n = 19), were included in the analysis. These groups were selected to represent common pediatric conditions with overlapping respiratory symptoms, such as cough, wheezing, and dyspnea, allowing for assessment of whether the observed metabolic alterations are specific to asthma or reflect a broader inflammatory response.

A key feature of the study design is its real-world clinical approach. Biological samples were obtained at initial presentation, prior to the establishment of a definitive diagnosis, from children presenting with similar respiratory symptoms. Following a comprehensive clinical evaluation, participants were subsequently classified into diagnostic groups. This study design enhances clinical relevance and enables evaluation of the potential of the biomarkers to differentiate asthma and asthma exacerbations from other respiratory conditions with similar presentations.

Participants aged 6–17 years were randomly and voluntarily recruited from patients who presented to the outpatient clinics of the Department of Child Health and Diseases at Sakarya University between March 2021 and March 2022. Ethical approval for all the procedures was obtained from Clinical Research Ethics Committee (ATADEK, 28 January 2021, decision no: 2021-02/07), and informed consent was obtained from all participants.

### 2.2. Study Protocol

As part of the initial medical examinations, a comprehensive medical history was taken from all participants, their physical examinations were conducted, and their blood samples were collected for routine laboratory tests. Blood specimens were collected at the time of diagnosis or during the initial clinical evaluation, adhering to appropriate preanalytical requirements. Following centrifugation, serum samples were stored at −80 °C until analysis.

During the COVID-19 pandemic, children presenting with overlapping respiratory and systemic symptoms such as dyspnea, tachypnea, chest retractions, wheezing, and gastrointestinal complaints were evaluated using a comprehensive diagnostic approach mandatory in routine clinical practice. The diagnostic workup included analysis of a standardized panel of laboratory biomarkers selected to assess the degree of inflammation, infectious burden, and systemic involvement. The following parameters were routinely evaluated: D-dimer, C-reactive protein (CRP), procalcitonin (PCT), ferritin, the ferritin-to-albumin ratio (FAR), aspartate aminotransferase (AST), the platelet-to-albumin ratio (PAR), and the neutrophil-to-lymphocyte ratio (NLR). Collectively, these markers served to facilitate differential diagnosis, evaluate disease severity, and guide the clinical decision-making process in this pediatric population. SARS-CoV-2 infection was confirmed via reverse transcription polymerase chain reaction (RT-PCR) tests performed on nasopharyngeal swab specimens, enabling classification of participants into the COVID-19 group.

In addition to assessment of conventional biomarkers, a panel of derived serological optimizing indicators (SDMA/ADMA, L-arginine/ADMA, NLR, FAR, and PAR) was incorporated into the analytical framework. These composite indicators were employed to integrate complementary biological pathways, minimize individual variability, and enhance the interpretability and comparative utility of biomarker profiles across study groups.

Laboratory parameters assessed included complete blood count, NLR, and routine inflammatory markers (CRP and PCT), along with vitamin B12, folic acid and 25(OH)D3 levels. Albumin, 25(OH)D3 D-dimer, AST, CRP, PCT, and ferritin levels were measured with Roche Cobas analyzers (Roche Diagnostics, Germany), and the NLR was determined using a Sysmex XN-9000 hematology analyzer (Sysmex Corporation, Kobe, Hyogo, Japan).

Methylated arginine metabolites were measured using a modified version of the analytical procedure previously described by Di Gangi et al. [15]. In this modified method, the samples were applied onto a 75 × 4.6 mm × 4 μm polar reversed-phase Phenomenex (Torrance, CA, USA) column and analyzed using Dionex high-performance liquid chromatography (HPLC) and Access MAX liquid chromatography with tandem mass spectrometry (LC-MS/MS) (Thermo Scientific, Waltham, MA, USA) devices, using “40 mM ammonium formate containing 3% formic acid (phase A) and acetonitrile (phase B)”. The flow rate was maintained at 300 μL/min. The chromatographic program was applied as follows: an initial isocratic phase with 10% B for 2 min, followed by a linear gradient from 10% to 30% B from 2 to 8 min, an isocratic phase at 30% B from 8 to 11 min, and an isocratic phase at 10% B from 11 to 20 min. Multiple reaction monitoring (MRM) parameters (m/z ratios of precursor and daughter ions) and optimal collision energy (CE) settings for each analyte were established through preliminary fragmentation studies by continuous infusion of 40 mmol/L stock solutions into the mass spectrometer. Ratios of m/z of precursor and daughter ions and CE for each analyte were determined as follows: 259.3–214.0 (25 V), 266.6–221.0 (15 V), 259.3–228.0 (15 V), 245.3–70.2 (15 V), 231.3–70.1 (15 V), 245.2–84.2 (24 V), for ADMA, ADMA-D7 (internal standard), SDMA, L-NMMA, arginine, and homoarginine, respectively. Additional conditions were as follows: capillary temperature: 210 °C; vaporizer temperature: 350 °C; sheath gas: 30 Arb, aux gas: 10 Arb, spray voltage: 3000 V; and positive ionization mode.

Serial dilutions of the respective stable isotypes were used as calibration standards. Dilutions of the standard solutions were as follows: 0.05, 0.1, 0.5, 1, and 2.4 μmol/L for ADMA, SDMA and homoarginine; 5, 10, 50, 100, 200, and 400 nmol/L for L-NMMA; and 5, 10, 50, 100, 200, and 400 μmol/L for arginine.

Intra- and inter-assay coefficients of variation (%) and recovery (%) values were <6.4%, <10.2% and 103% for ADMA; <7.1, <12.1% and 96% for SDMA; <7.8, <14.2% and 96% for L-NMMA; <5.1, <7.8% and 98% for arginine; and <6.7, <11.6% and 105% for homoarginine, respectively. Limit of quantitation (LoQ) values were determined to be 0.02 μmol/L for ADMA, 0.04 μmol/L for SDMA, 9.3 nmol/L for L-NMMA, 3.1 μmol/L for arginine, and 0.05 μmol/L for homoarginine.

Aliquots of 200 μL, from each serum sample, were placed in a fresh tube and mixed with 100 μL of ADMA-D7 (1 μmol/L internal standard) and 1 mL of methanol. The tubes were vortexed for 1 min and centrifuged at 13,000 rpm for 10 min at room temperature. The supernatants were then transferred to new unused tubes, and the solvent was evaporated under nitrogen at 65 °C. Subsequently, 200 μL of the derivatization solution, prepared by mixing 19 volumes of butanol with 1 volume of acetyl chloride, was added. The samples were incubated at 65 °C for 30 min for derivatization. After incubation, the samples were evaporated under nitrogen. Pellet remnants were dissolved with 200 μL of “10% methanol–0.1% formic acid” solution. Volumes of 20 μL of the dissolved samples were transferred to autosampler vials for LC–MS/MS analysis. Samples that were separated chromatographically in the analytical column were then transferred to the mass spectrometer. Selected ions (precursor ions) were broken down into fragments (daughter ions) in the collision cell of the tandem mass spectrometer, enabling more definitive measurements.

### 2.3. Statistical Analyses

An a priori power analysis was conducted using the G-power software (version 3.1.9.7) to determine adequacy of the study sample. Based on the estimated effect size, an alpha level of 0.05, a statistical power of 95%, and a total sample size of 154 participants were considered sufficient to detect differences among six study groups.

Descriptive statistics were presented in the form of median, minimum, maximum, frequency, and percentage values depending on the nature of the variables (Table 1). The distributions of blood test results across study groups, summarized using median values and interquartile ranges, are shown in Table 2. As most continuous variables were not normally distributed, group comparisons were performed using the Mann–Whitney U test.

Based on the results of the univariate analysis using the Mann–Whitney U test, variables demonstrating statistical significance were considered for inclusion in the multivariable model. Since ADMA and the SDMA/ADMA ratio demonstrated substantial intercorrelation, only the variable with the greater discriminatory capacity between groups was retained to prevent multicollinearity and ensure model stability. For the regression analyses, disease status was considered as a binary dependent variable. In the asthma model, people with a diagnosis of asthma were labeled as 1, and all other participants were labeled as 0. Correspondingly, in the asthma exacerbation model, children with an acute asthma attack were labeled as 1, and all other participants were labeled as 0. Serum concentrations of citrulline, ornithine, and L-arginine and the SDMA/ADMA ratio were considered as independent variables. Binary logistic regression analysis was performed to evaluate the independent associations between biochemical parameters and disease status, allowing for simultaneous evaluation of multiple biomarkers. Separate regression models were constructed for asthma and asthma exacerbation. Odds ratios (ORs) with 95% confidence intervals (CIs) were calculated. Model fit was assessed using the Hosmer–Lemeshow goodness-of-fit test. A *p*-value < 0.05 was considered statistically significant. Receiver operating characteristic (ROC) curve analysis was performed to assess the discriminative ability of the regression models. Predicted probabilities derived from the logistic regression models were used to construct ROC curves, and the area under the curve (AUC) was calculated. Optimal cut-off values were determined based on the Youden index, maximizing the combined sensitivity and specificity.

## 3. Results

### 3.1. Baseline Characteristics

Descriptive statistics for demographic characteristics and laboratory parameters are summarized in Table 1. The median age was 12.5 years (range: 6–17 years) in the asthma group and 11.0 years (range: 6–17 years) in the asthma exacerbation group. No statistically significant difference in age distribution was observed among the study groups (*p* = 0.062). Similarly, the distribution of gender among study participants did not differ significantly between groups.

### 3.2. Group Comparisons of Laboratory Parameters

Intergroup comparisons of laboratory parameters were performed using the Kruskal–Wallis test due to the non-normal distribution of the data. The results are presented in Table 2. Significant differences were detected between groups for D-dimer, PCT, ferritin, FAR, AST, PAR, NLR, ornithine, ADMA, L-arginine, citrulline, and the SDMA/ADMA ratio (*p* < 0.05).

Parameters related to arginine metabolism demonstrated distinct distribution patterns across clinical groups. ADMA levels were significantly higher in asthma patients relative to the control group. L-arginine levels were lowest in the asthma exacerbation group, which also exhibited the lowest L-arginine/ADMA ratio, whereas the highest values were observed in the control group. Similarly, the SDMA/ADMA ratio was highest in the control group and lowest in the controlled asthma group. Moreover, the levels of citrulline were significantly different in all study groups, and the lowest concentrations were detected in the patients with asthma exacerbations, whereas the highest levels were observed in the control group.

### 3.3. Regression and ROC Analyses

We applied multivariable logistic regression as the main statistical method used in addition to descriptive group comparisons to examine the independent relationships between biomarkers linked to arginine–NO metabolism and asthma exacerbation. While nonparametric tests were used to explore unadjusted differences across groups, regression analysis was chosen to account for potential confounding factors and to better reflect clinically meaningful distinctions between asthma exacerbation and stable disease. Specifically, the SDMA/ADMA ratio (*p* = 0.001) and ornithine (*p* = 0.003), citrulline (*p* < 0.0001), and arginine levels (*p* < 0.0001) were entered into the binary logistic regression analysis to evaluate their independent associations with the outcome variables. We believe that use of this additional analytical method increases the comprehension and clinical value of our findings.

Binary logistic regression analysis was performed to discover biomarkers separately related with asthma (Table 3). After adjustment for multiple covariates, the SDMA/ADMA ratio showed inverse associations with controlled asthma. Other variables included in the model did not demonstrate statistically significant associations with asthma.

**Figure 2:**
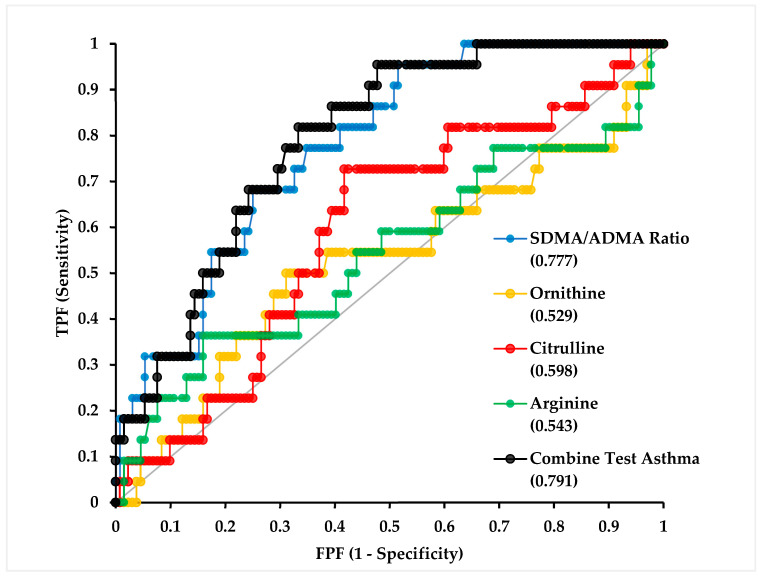
ROC analysis for controlled asthma group. Abbreviations: TPF: True Positive Fraction; FPF: False Positive Fraction; SDMA: symmetric dimethylarginine; ADMA: asymmetric dimethylarginine.

Receiver operating characteristic (ROC) curve analyses were performed to evaluate the discriminative performance of arginine-related metabolites as variables included in the binary logistic regression model, which made comparisons among children with controlled asthma (n = 22) and a combined comparison group comprising pneumonia, URTI, asthma exacerbation, COVID-19, and healthy controls (n = 132), with the results presented in Figure 2.

Among the evaluated biomarkers, the SDMA/ADMA ratio demonstrated moderate discriminative ability in this dataset, with an AUC of 0.777 (95% CI: 0.685–0.868, *p* < 0.0001), as shown in Table 4. The combined test model yielded relatively higher discriminative performance, with an AUC of 0.791 (95% CI: 0.705–0.878, *p* < 0.0001), suggesting that the concurrent evaluation of these markers may offer modest improvement in classification accuracy within the present study population.

Overall, these findings indicate that the SDMA/ADMA ratio, both individually and as part of a combined marker model, was independently associated with controlled asthma status within this cross-sectional dataset.

Binary logistic regression analysis revealed that among the four variables examined, the SDMA/ADMA ratio was independently associated with asthma exacerbation status in this dataset, indicating that higher SDMA/ADMA ratio values were associated with increased odds of belonging to the exacerbation group (Table 5). L-arginine level demonstrated inverse associations with asthma exacerbation. Other inflammatory and hematological markers included in the model did not retain statistical significance after adjustment.

**Figure 3:**
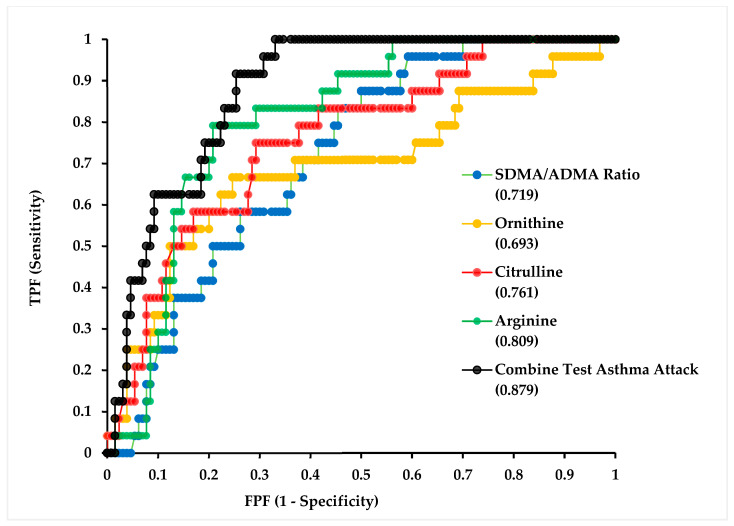
ROC analysis for the asthma exacerbation group. Abbreviations: TPF: True Positive Fraction; FPF: False Positive Fraction; SDMA: symmetric dimethylarginine; ADMA: asymmetric dimethylarginine.

ROC curve analyses were performed to evaluate the discriminative performance of individual metabolites and their combined model in identifying asthma exacerbation within the present dataset (Figure 3). The SDMA/ADMA ratio demonstrated a fair discriminative ability with an AUC of 0.719 (95% CI: 0.625–0.813, *p* < 0.0001). L-Arginine exhibited a higher discrimination within this sample (AUC: 0.809, 95% CI: 0.730–0.887, *p* < 0.0001). When all variables were incorporated into a multivariable combined test model, the AUC increased to 0.879 (95% CI: 0.823–0.936, *p* < 0.0001), suggesting that the concurrent consideration of these markers may offer improved classification accuracy in the present study population.

Table 6 summarizes the cut-off value, 95% CI, specificity, and specificity values for the ROC analysis for the asthma exacerbation group. ROC analysis identified an optimal cut-off value of 0.535 for the SDMA/ADMA ratio. At this threshold, the sensitivity was 70.8%, while the specificity was 61.5%, indicating a moderate ability to correctly classify individuals with and without the condition. The positive likelihood ratio (LR+) was 1.84, suggesting a limited increase in the probability of disease presence when the test is positive, whereas the negative likelihood ratio (LR−) was 0.47, indicated a modest reduction in disease probability when the test is negative. The corresponding odds ratio was 3.89, reflecting a moderate association between higher SDMA/ADMA ratio values and the outcome of interest. Overall, these findings suggest that the SDMA/ADMA ratio has moderate discriminative performance and may provide supportive, but not definitive, diagnostic value.

For arginine, ROC curve analysis demonstrated good discriminative performance (AUC = 0.809, 95% CI: 0.730–0.887, *p* < 0.0001), as shown in Table 6. The optimal cut-off value of 56.4, determined using the Youden index, corresponded to a sensitivity of 79.2% and a specificity of 79.2%, with a positive likelihood ratio of 3.81, a negative likelihood ratio of 0.26, and a diagnostic odds ratio of 14.4, indicating moderate-to-good diagnostic utility within the present dataset.

The combined biomarker model yielded the highest discriminative performance among all evaluated markers (AUC = 0.879, 95% CI: 0.823–0.936, *p* < 0.0001). At the optimal cut-off of 0.156, the model achieved a sensitivity of 91.7% and a specificity of 74.6%, with a positive likelihood ratio of 3.61, a negative likelihood ratio of 0.11, and a diagnostic odds ratio of 32.3. The notably low negative likelihood ratio suggests that a negative combined test result may be particularly informative in ruling out asthma exacerbation within this study population. However, these findings require external validation before broader clinical interpretation.

**Figure 4:**
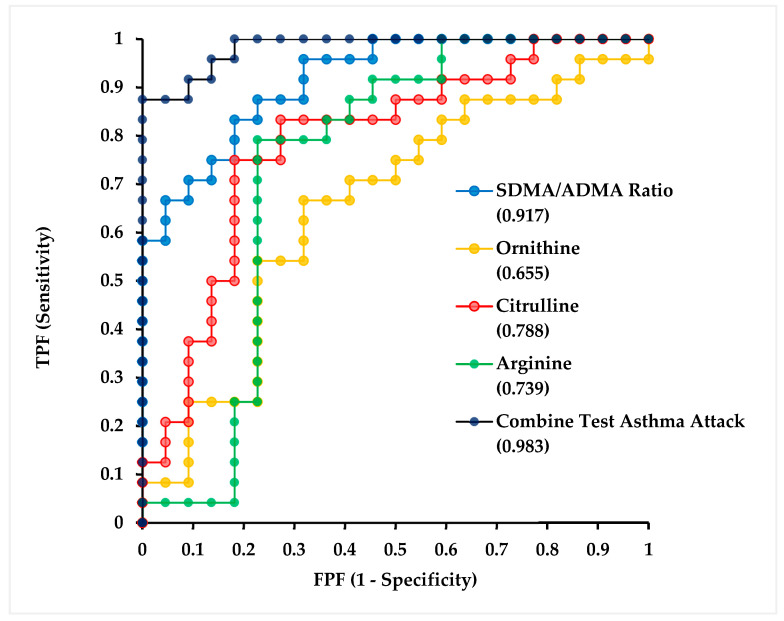
ROC analysis of biomarkers in distinguishing asthma exacerbation from controlled asthma. Abbreviations: TPF: True Positive Fraction; FPF: False Positive Fraction; SDMA: symmetric dimethylarginine; ADMA: asymmetric dimethylarginine.

ROC curve analyses were performed to evaluate the discriminative performance of individual metabolites and their combined model in identifying and distinguishing asthma exacerbation from controlled asthma (Figure 4). Table 7 summarizes the cut-off value, 95% CI, specificity, and specificity values for the ROC analysis of biomarkers used to distinguish asthma exacerbations from controlled asthma.

The combined model demonstrated excellent diagnostic accuracy in distinguishing asthma attack from controlled asthma, with an AUC of 0.983 (95% CI: 0.958–0.983), a sensitivity of 87.5%, and a specificity of 95.5% at the optimal cut-off value of 0.759, as determined by the Youden index. The perfect specificity observed at the optimal threshold should therefore be interpreted with caution, and external validation in larger cohorts is warranted.

## 4. Discussion

This study integrates the existing literature with arginine–NO metabolism in children with controlled asthma and during acute asthma exacerbation and highlights that different and context-specific biomarker profiles become apparent when multivariable analytical methods are used. The results indicate that biomarkers associated with this pathway behave differently in chronic disease and during acute exacerbation, thus reflecting the pathophysiological heterogeneity of asthma.

Nonparametric group comparisons revealed significant differences in numerous inflammatory and metabolic markers between asthma (controlled and exacerbation phase) and other study groups in terms of numerous inflammatory and metabolic markers. As illustrated in Table 2, the SDMA/ADMA ratio reached its highest values in the control group and its lowest in the controlled asthma group. This pattern suggests that the balance between SDMA and ADMA is disrupted even during clinically stable phases of asthma, a finding that underscores the persistent dysregulation of arginine methylation in this chronic condition [16,17,18,19]. The physiological rationale for examining the SDMA/ADMA ratio lies in the fundamentally distinct metabolic fates of these two isomers. Although SDMA does not directly inhibit NOS, it reduces intracellular L-arginine availability by competing for cellular uptake via cationic amino acid transporter-1 (CAT1), thereby indirectly impairing NO synthesis [20,21,22]. ADMA is predominantly cleared through enzymatic degradation by dimethylarginine dimethylaminohydrolase (DDAH), an enzyme susceptible to inactivation by oxidative stress. SDMA is eliminated almost exclusively via renal excretion [23]. Consequently, the SDMA/ADMA ratio does not merely reflect the absolute levels of either molecule in isolation but rather captures the dynamic balance between global arginine methylation burden (reflected by SDMA) and DDAH-dependent metabolic clearance capacity (reflected by ADMA) [17]. Therefore, an elevated SDMA/ADMA ratio may indicate a state in which DDAH activity is relatively preserved or compensated, while overall protein turnover and inflammatory methylation load are disproportionately increased, which could be interpreted as a potentially consistent biochemical signature with increased systemic inflammation, characteristic of asthma exacerbation. Conversely, healthy individuals appear to maintain a relatively preserved and more favorable equilibrium in arginine-methylation-related pathways, suggesting that the biochemical homeostasis governing methylarginine metabolism may be fundamentally altered in asthmatic patients, irrespective of their symptomatic status [24].

In multivariable binary logistic regression analysis, biomarker metabolites of SDMA/ADMA and L-arginine biomarker metabolites showed different associations with disease status (Table 3 and Table 5). The divergent patterns observed across L-arginine pathway metabolites, including SDMA/ADMA associations with asthma exacerbation(s) and the inverse behavior of L-arginine during asthma exacerbation conditions, align with the notion that arginine metabolism undergoes state-dependent remodeling, potentially implicating distinct physiological pathways in the transition between stable and exacerbated disease [25].

Citrulline and L-arginine concentrations followed a consistent gradient across groups, reaching their lowest levels in patients experiencing acute asthma exacerbation and their peak in healthy controls, a pattern that can lend support to the view that asthma exacerbations constitute a distinct metabolic phenotype defined by profound dysregulation of arginine metabolism [21,22,26,27,28].

To further evaluate the discriminative utility of the identified biomarkers, receiver operating characteristic (ROC) analysis was performed for the asthma exacerbation group using a combined test approach, as shown in Table 6. The combined model demonstrated strong diagnostic performance, yielding an area under the curve (AUC) of 0.879 (95% CI: 0.823–0.936), which reflects excellent discriminative ability in distinguishing the exacerbation group from the remaining study groups. At the optimal cut-off value of 0.156, the model achieved a sensitivity of 91.7% and a specificity of 74.6%, indicating that the combined test correctly identified the large majority of exacerbation cases while maintaining an acceptable rate of false positives. The positive likelihood ratio of 3.61 suggests that a positive test result is approximately 3.6 times more likely to be observed in a patient with an asthma exacerbation than in one without, while the negative likelihood ratio of 0.11 indicates that a negative test result substantially reduces the post-test probability of exacerbation, conferring strong rule-out value. Notably, the odds ratio of 32.3 implies that individuals testing positive by the combined model are over 32 times more likely to belong to the exacerbation group compared to those testing negative, further highlighting the clinical discriminative power of this multimarker approach. Taken together, these findings suggest that the combined assessment of arginine pathway metabolites may offer clinically meaningful added value in differentiating active asthma exacerbation from other disease states and warrants its validation in larger prospective cohorts.

To assess the capacity of individual arginine pathway metabolites and their combination to discriminate asthma exacerbation from controlled asthma within this dataset, ROC analysis was performed for each biomarker as well as for a combined multimarker model (Table 7). These analyses should be interpreted in the context of the cross-sectional study design, and the performance metrics reported here reflect associations observed within this specific dataset rather than validated predictive accuracy. Among the individual biomarkers, the SDMA/ADMA ratio demonstrated the strongest standalone discriminative performance, yielding an AUC of 0.917 (95% CI: 0.842–0.991). At a cut-off value of 0.468, it achieved a sensitivity of 83.3% and a specificity of 81.8%, with a positive likelihood ratio of 4.58, a negative likelihood ratio of 0.20, and an odds ratio of 22.5 within this sample (Table 7). These figures suggest that the SDMA/ADMA ratio may significantly make the distinction between exacerbation and controlled disease states in this dataset, supporting its further investigation as a potential marker of acute disease activity within the L-arginine–NO pathway [29,30].

The combined test integrating all four metabolites yielded an AUC of 0.983 (95% CI: 0.958–0.983), indicating strong discriminative performance within this dataset. At a cut-off value of 0.759, the model achieved a sensitivity of 87.5% and a specificity of 95.5%, with a positive likelihood ratio of 19.25, a negative likelihood ratio of 0.13, and an odds ratio of 147 (Table 7). While these figures indicate strong discriminative performance within this dataset, they warrant cautious interpretation given the exploratory and cross-sectional nature of the study, the relatively modest subgroup sample sizes, and the absence of external validation. High discriminative performance in small samples carries a recognized risk of overfitting, whereby the model may capture dataset-specific noise rather than a generalizable biological signal. Taken together, these findings suggest that arginine pathway metabolites, particularly the SDMA/ADMA ratio, are independently associated with asthma exacerbation status in this dataset and that their combined assessment may warrant further evaluation in larger, prospectively designed studies before any clinical utility of assessing these metabolites can be established.

### Strengths and Limitations

While the present study has several important strengths, certain limitations should be acknowledged. First, although recruitment included all eligible patients presenting during the predefined study period at a single tertiary referral center, the relatively small sample size within individual subgroups may limit the generalizability of the findings and should be considered when interpreting the results.

Beyond sample size, several potential confounders merit consideration. Patients were receiving heterogeneous treatment regimens, including both monotherapy and combination therapies with variable durations, which may have influenced biomarker levels. Furthermore, variables known to affect SDMA and ADMA concentrations, including body mass index (BMI), nutritional status, and renal function, could not be fully controlled despite efforts to standardize clinical conditions [31,32]. Multicollinearity between ADMA and the SDMA/ADMA ratio was identified a priori; accordingly, only one of these correlated variables was retained in the final model to reduce variance inflation and improve parsimony, though the possibility of residual confounding cannot be entirely excluded.

The study was additionally conducted under the constraints of the COVID-19 pandemic, which significantly affected patient recruitment and follow-up. Hospital admissions were restricted during this period, and many patients with well-controlled asthma were managed via remote follow-up rather than in-person visits, limiting their eligibility for inclusion. Furthermore, some initially eligible participants were excluded due to underlying chronic diseases identified during screening. These factors collectively contributed to the assessment of a relatively small sample size. Biomarker performance in a single-center, cross-sectional study may also be overestimated, and the absence of external validation limits the immediate applicability of the findings in clinical practice. While the high AUC value observed for the combined model is promising, it may partially reflect dataset-specific noise rather than a truly generalizable biological signal and should therefore be interpreted with caution.

Despite these limitations, the study offers several notable strengths. It provides a comprehensive comparison of clinically overlapping respiratory diseases in a pediatric population, incorporating both intra-disease variations (controlled versus exacerbation phases of asthma) and inter-disease comparisons, alongside the simultaneous assessment of a broad panel of methylated arginine metabolites and inflammatory biomarkers. A key methodological strength is its real-world clinical design. The biological samples were collected at the time of initial presentation, prior to definitive diagnosis, after which participants were allocated into diagnostic groups, including COVID-19, pneumonia, upper respiratory tract infection, controlled asthma, and asthma exacerbation following comprehensive clinical evaluation. This approach enhances the clinical relevance of the findings by reflecting routine pediatric practice and supports the evaluation of these biomarkers as potentially independently associated markers in differentiating conditions with similar initial presentations as well as in characterizing stage-specific differences within asthma.

Although established clinically significant biomarkers such as CRP, PCT, and D-dimer are already widely used in the differential diagnosis of respiratory conditions primarily reflecting inflammation or infection, arginine-related metabolites may provide complementary information related to nitric oxide metabolism and endothelial function. Nevertheless, further studies directly comparing their diagnostic performance and evaluating their incremental value in multivariable clinical models are warranted. To the best of our knowledge, this is one of the few pediatric studies investigating these biomarkers across multiple respiratory conditions, and the findings may provide a foundation for future multicenter studies aimed at developing biomarker-based diagnostic and prognostic strategies in pediatric respiratory diseases.

## 5. Conclusions

Taken together, these findings suggest that commonly used biomarkers, including ADMA, D-dimer, CRP, and PCT, may have limited discriminative value when interpreted in isolation for defining different stages of asthma. Importantly, the results highlight that biomarkers involved in nitric oxide metabolism exhibit distinct behavior during acute exacerbations compared with stable disease, underscoring the dynamic nature of metabolic regulation in asthma.

Multivariable regression analysis revealed distinct independent associations within this dataset: elevated ADMA levels were independently associated with controlled asthma, whereas SDMA/ADMA ratios appeared to be linked to asthma exacerbation. These findings suggest that the SDMA/ADMA ratio may have potential clinical utility in distinguishing asthma exacerbation from other respiratory conditions. However, evaluating the SDMA/ADMA ratio solely as a diagnostic marker may represent a limited perspective. Therefore, its mechanistic implications and its potential therapeutic relevance could offer a more comprehensive understanding of this condition. As ADMA inhibits NOS and SDMA impairs cellular L-arginine uptake through competition at the CAT1 transporter level, an elevated SDMA/ADMA ratio may reflect a shift forward dysfunctional NO metabolism during exacerbation, presenting a pathway that could be an arena for targeted intervention. ROC analysis further demonstrated that while individual markers, most notably L-arginine, exhibited meaningful discriminatory performance, the combined model that included all five parameters produced the highest overall diagnostic accuracy, highlighting the complementary nature of these metabolites when evaluated collectively. Taken together, these findings suggest that the arginine–nitric oxide pathway differs substantially between these two clinical states, highlighting its potential role not only as a discriminative biochemical axis in the spectrum of asthma severity, but also as a mechanistically ground target for future therapeutic strategies.

However, although the diagnostic performance appears promising, its clinical applicability remains limited without its external validation and comparison with established clinical and laboratory markers.

Overall, these findings suggest that the evaluated biomarkers were independently associated with asthma exacerbation status within this cross-sectional dataset. However, in the absence of external validation, these results should be interpreted with caution, as the observed discriminative performance may not be generalized beyond the current sample.

These observations may carry meaningful implications for understanding the mechanistic underpinnings of disease control versus acute exacerbation and warrant conduction of further investigations in larger, prospectively designed cohorts.

## Figures and Tables

**Table 1:** Descriptive characteristics of the study participants.

Groups/ Variables	Controlled Asthma (n: 22)	Asthma Exacerbation (n: 24)	URTI (n: 38)	Pneumonia (n: 15)	COVID-19 (n: 36)	Control (n: 19)	*p*
Age (year), range	12.5 (6.0–17.0)	11.0 (6.0–17.0)	13.0 (6.0–17.0)	9.0 (6.0–16.0)	14.5 (6.0–17.0)	9.0 (6.0–17.0)	0.062
Gender (male), n (%)	11 (50)	15 (62)	13 (35)	5 (33)	17 (47)	7 (36)	

Abbreviation: URTI: upper respiratory tract infection.

**Table 2:** Group comparisons of laboratory parameters.

Groups/Variables	Controlled Asthma (n: 22)	Asthma Exacerbation (n: 24)	URTI (n: 38)	Pneumonia (n: 15)	COVID-19 (n: 36)	Control (n: 19)	*p*
**D-dimer** **(μg/L FEU)**	279 (258.8–367.3)	517.5 ** **(484.3–549.5)**	201.5 (129.5–301)	596 * **(386–681)**	850 * **(617.5–1420)**	210 (155.5–238.5)	<0.0001 *
**CRP** **(mg/L)**	4.00 (3.30–5.20)	4.00 (2.08–7.13)	1.15 (0.53–3.25)	35.1 * **(22.0–71.1)**	2.7 (1.3–7.9)	1.50 (0.95–2.0)	<0.001 *
**Procalcitonin (ng/mL)** **(PCT)**	0.04 (0.03–0.05)	0.05 (0.03–0.06)	0.03 (0.01–0.04)	1.10 * **(0.16–2.13)**	0.07 (0.04–0.22)	0.03 (0.02–0.07)	0.003 *
**Ferritin** **(ng/mL)**	34.1 (25.7–44.8)	50.6 * **(45.6–58.5)**	29.6 (19.7–42.2)	72.9 * **(60–134.1)**	67.6 * **(57.8–96.6)**	27.5 (19.3–38.5)	<0.001 *
**FAR** **(ferritin/albumin)**	7.0 (5.0–8.8)	13.0 ** **(11.8–14.3)**	7.0 (4.0–9.8)	18.0 * **(15.0–31.5)**	15.0 * **(12.0–23.3)**	6.0 (4.0–9.5)	<0.001 *
**AST** **(U/L)**	19.0 (17.0–24.5)	28.0 ** **(24.5–31.5)**	24.0 (20.0–29.0)	27.0 * **(22.5–32.5)**	20.0 (17.8–28.3)	22.7 (18.3–26.5)	<0.0001 *
**PAR** **(platelet/albumin)**	66,666 (60,725.5–80,534.8)	85,137.5 ** **(63,599–99,437.5)**	63,395.5 (47,012.5–77,881.8)	70,426 (61,120–86,506.5)	57,468.5 * **(48,028.5–69,634)**	77,049 (58,966.5–87,878.5)	<0.001 *
**NLR** **(neutrophil/lymphocytes)**	1.87 (1.36–2.54)	2.31 ** **(1.71–3.66)**	1.81 (1.13–2.90)	4.89 * **(4.07–6.47)**	2.06 (1.27–3.02)	0.89 (0.54–1.55)	<0.001 *
**SDMA** **(μmol/L)**	0.34 (0.28–0.44)	0.39 (0.33–0.48)	0.34 (0.27–0.50)	0.44 (0.33–0.53)	0.32 (0.26–0.41)	0.42 (0.33–0.48)	0.1000
**ADMA** **(μmol/L)**	0.82 ** **(0.65–1.5)**	0.60 (0.52–0.78)	0.77 (0.56–1.2)	0.66 (0.52–0.92)	0.68 (0.53–0.98)	0.57 (0.51–0.69)	0.007 *
**SDMA/ADMA**	0.35 ** **(0.29–0.44)**	**0.65** **(0.50–0.84)**	0.39 (0.31–0.56)	0.67 (0.46–0.78)	0.45 (0.38–0.58)	0.74 (0.60–0.88)	<0.0001 *
**Ornithine**	72.6 (40.1–96.1)	39.0 ** **(25.5–73.3)**	63.1 (40.3–85.8)	61.9 (40.3–74.3)	67.8 (48.9–86.9)	64.2 (49.6–81.0)	0.01 *
**L-Arginine** **(μmol/L)**	90.2 (58.2–135.1)	46.4 ** **(38.8–55.5)**	78.2 (48.7–119.2)	64.9 (59.3–88.6)	109.8 (71.0–124.8)	112.5 (88.0–127.3)	<0.001 *
**L-Arginine/ADMA**	84.0 * **(70.0–106.3)**	75.0 * **(51.8–122.3)**	84.0 * **(70.0–130.3)**	89.0 * **(75.5–143.5)**	122.0 * **(101.0–204.5)**	195.0 (167.0–228.0)	<0.001 *
**Citrulline** **(μmol/L)**	30.8 * **(23.5–38.4)**	20.2 * **(19.5–22.2)**	26.4 * **(21.2–39.0)**	24.2 * **(21.9–29.3)**	21.7 * **(21.0–27.6)**	50.6 (39.3–68.9)	<0.001 *

Data are expressed as median values and 25–75th percentiles. ** Mann–Whitney’s U test was used for pairwise comparison, * significant difference at the 0.05 level. ADMA: asymmetric dimethylarginine, CRP: C-reactive protein, SDMA: symmetric dimethylarginine.

**Table 3:** Binary logistic regression analysis for the controlled asthma group.

Variables	B (Value)	Pr > Chi^2^	Odds Ratio (OR)	Odds Ratio Lower Limit (95%)	Odds Ratio Upper Limit (95%)
SDMA/ADMA	−7.538	**<0.001**	0.001	0.001	0.028
Ornithine	0.007	0.307	0.993	0.979	1.007
Citrulline	0.037	0.111	1.038	0.991	1.087
L-Arginine	−0.001	0.871	0.999	0.985	1.013

Abbreviations: ADMA: asymmetric dimethylarginine, SDMA: symmetric dimethylarginine.

**Table 4:** ROC analysis results for the controlled asthma group.

	AUC	95% CI	Cut-Off Value	Sensitivity	Specificity	Likelihood Ratio (+)	Likelihood Ratio (−)	Odds Ratio
SDMA/ADMA Ratio	0.777	0.685–0.868	0.393	0.682	0.750	2.73	0.42	6.429
Ornithine	0.529	0.381–0.677	69.38	0.545	0.606	1.38	0.75	1.846
Citrulline	0.598	0.472–0.724	27.28	0.727	0.583	1.75	0.47	3.733
L-Arginine	0.543	0.392–0.693	80.96	0.591	0.508	1.20	0.81	1.486
Combined Test	0.791	0.705–0.878	0.134	0.818	0.667	2.45	0.27	9.0

Abbreviations: ROC: receiver operating characteristic analysis; SDMA: symmetric dimethylarginine; ADMA: asymmetric dimethylarginine.

**Table 5:** Binary logistic regression analysis for asthma exacerbation group.

Variables	B (Value)	Pr > Chi^2^	Odds Ratio (OR)	Odds Ratio Lower Limit (95%)	Odds Ratio Upper Limit (95%)
SDMA/ADMA	4.273	**0.001**	71.718	5.787	888
Ornithine	0.002	0.818	1.002	0.988	1.016
Citrulline	−0.063	0.212	0.939	0.850	1.037
L-Arginine	−0.032	**0.011**	0.969	0.945	0.993

Abbreviations: SDMA: symmetric dimethylarginine; ADMA: asymmetric dimethylarginine.

**Table 6:** ROC analysis results for the asthma exacerbation group.

	AUC	95% CI	Cut-Off Value	Sensitivity	Specificity	Likelihood Ratio (+)	Likelihood Ratio (−)	Odds Ratio
SDMA/ADMA Ratio	0.719	0.625–0.813	0.535	0.708	0.615	1.84	0.47	3.89
Ornithine	0.693	0.562–0.825	42.1	0.667	0.754	2.71	0.44	6.125
Citrulline	0.761	0.659–0.862	23.8	0.792	0.623	2.10	0.33	6.282
L-Arginine	0.809	0.730–0.887	56.4	0.792	0.792	3.81	0.26	14.4
Combine Test	0.879	0.823–0.936	0.156	0.917	0.746	3.61	0.11	32.3

Abbreviations: AUC: area under the curve; ROC: receiver operating characteristic; SDMA: symmetric dimethylarginine; ADMA: asymmetric dimethylarginine.

**Table 7:** Results of ROC analysis of biomarkers used to distinguish asthma exacerbations from controlled asthma.

	AUC	95% CI	Cut-Off Value	Sensitivity	Specificity	Likelihood Ratio (+)	Likelihood Ratio (−)	Odds Ratio
SDMA/ADMA	0.917	0.842–0.991	0.468	0.833	0.818	4.58	0.20	22.5
Ornithine	0.655	0.492–0.819	43.9	0.667	0.682	2.10	0.49	4.286
Citrulline	0.788	0.651–0.925	21.9	0.750	0.818	0.31	0.75	13.5
L-Arginine	0.739	0.574–0.903	56.4	0.792	0.773	3.48	0.27	12.9
Combine Test	0.983	0.958–1.000	0.759	0.875	0.955	19.25	0.13	147

Abbreviations: AUC: area under the curve; ROC: receiver operating characteristic; SDMA: symmetric dimethylarginine; ADMA: asymmetric dimethylarginine.

## Data Availability

The datasets generated and analyzed during the current study are not publicly available but are available from the corresponding author on reasonable request.

## Abbreviations

The following abbreviations are used in this manuscript:

ADMA	Asymmetric dimethylarginine
CAT1	Cationic amino acid transporter-1
CE	Collision energy
CI	Confidence interval
CRP	C-reactive protein
eNOS	Endothelial nitric oxide synthase
LC MS/MS	Liquid chromatography with tandem mass spectrometry
LNMMA	NG-monomethyl-L-arginine
MS	Mass spectrometry
NO	Nitric oxide
NOS	Nitric oxide synthase
OR	Odds ratio
PCT	Procalcitonin
PRMTs	Protein arginine methyltransferases
SDMA	Symmetric dimethylarginine
